# Germanium metasurface assisted broadband detectors

**DOI:** 10.1515/nanoph-2023-0116

**Published:** 2023-05-11

**Authors:** Torgom Yezekyan, Vladimir A. Zenin, Martin Thomaschewski, Radu Malureanu, Sergey I. Bozhevolnyi

**Affiliations:** Centre for Nano Optics, University of Southern Denmark, Campusvey 55, 5230, Odense, Denmark; The George Washington University, 800 22nd St NW, Washington, 20052, Washington, DC, USA; DTU Electro, Technical University of Denmark, Oersteds Plads, Bldg 345V Rm 173, 2800, Kgs Lyngby, Denmark

**Keywords:** germanium, metasurfaces, photodetectors

## Abstract

The demand on broadband near-infrared photodetections with high responsivity is becoming increasingly eminent; however its realization remains a significant technological challenge. Here we design, fabricate, and characterize a broadband Ge photodetector (1000–1600 nm), composed of densely packed 230-nm-thick Ge disks of different diameters (255 nm, 320 nm, and 500 nm), placed on top of a 105-nm-thin Ge layer. Using experimentally measured and calculated transmission and absorption spectra, we demonstrate that the absorption and detector responsivity are increased by nearly 2 orders of magnitude, compared to the unstructured Ge photodetector, due to the excitation of magnetic dipole resonances in Ge disks, while preserving a relatively low dark current. Our approach is simple and can be easily adapted to other semiconductor material platforms and operation wavelengths to enable performance improvements of broadband photodetector devices.

## 1 Introduction

Photodetectors (PD), devices that convert electromagnetic radiation into electrical currents and thereby optical into electrical signals, play a fundamental role in modern industrial, scientific, biomedical, information, and communication applications [1–3]. The most efficient way of photodetection is to use semiconductor-based PDs with absorbed photons generating electron–hole pairs, which are separated by external or internal electric fields and collected into measurable photocurrents. For this process to occur, the photon energy should exceed the energy band gap (BG) of the semiconductor. The excess is however should not be too large as it transforms mainly into the heat. Silicon (Si) with the BG of ∼1.1 eV [4] is an unbeaten leader among semiconductors to be used in PDs operating in the visible region [5]. The situation is not so simple in the near-infrared range (NIR), where the BG required should be ∼0.5 eV. Therefore, most of modern NIR PDs are based on III–V semiconductors: indium gallium arsenide (InGaAs, BG ∼ 0.75 eV), indium antimonide (InSb, BG ∼ 0.17 eV), germanium (Ge, BG ∼ 0.66 eV), etc. [4, 6]. These detectors require rather complex manufacturing accompanied by high cost, and, most importantly, are incompatible with the CMOS technologies. Herewith, Ge seems to be a very good candidate for NIR PDs, mainly due to its relatively simple manufacturing (and, thus, lower costs) [7], and is therefore also often chosen in many laboratories investigating novel designs of NIR Ge-based PDs [8–10].

There are several trends one would usually follow to optimize the PD performance: increasing the photocurrent, decreasing the response time, decreasing the dark current, decreasing the noise, etc. All above can benefit from reduction of both the lateral semiconductor size and its thickness (the photocurrent is enhanced for a shorter transit time of the photogenerated carriers). The latter, however, results in a reduced absorption and, thus, in an unwanted decrease of the photocurrent and the PD efficiency, when the thickness becomes smaller than the light penetration depth. Therefore, various approaches are investigated to increase the light absorption while maintaining small PD thicknesses, such as band structural engineering, enhancement by plasmonic nanostructures etc. [2, 11], [12], [13], [14], [15]. However, the increased responsivity comes usually at the price of increased dark currents, let alone the fact that Ge PDs already have rather high dark currents attributed to dislocations in Ge formed during the growth [16].

The electric field inside materials and, thus, the absorption, can be boosted by using optical resonances. It was shown that lattice resonances caused by the normally incident free-space light being redirected by periodic nanostructures to propagate in-plane inside thin layer of a semiconductor can strongly enhance the otherwise very weak absorption in Ge [17, 18]. However, the optical bandwidth and angular sensitivity are very narrow, while fabrication requirements with respect to the nanostructure dimensions and periodicity are high. Alternatively, one can employ resonances of individual nanoparticles, and achieve broad absorption bandwidth by combining nanoparticles of different size [19].

Here, we boost the absorption in a 105-nm-thin Ge by covering it with 230-nm-thick Ge disks of three different diameters (255, 320, and 500 nm). The disk shape was chosen for simplicity, but its size and thickness were optimized to maximize absorption at three design wavelengths: 1200, 1375, and 1575 nm, which combined cover the whole design NIR range of 1000–1600 nm. There is nearly a free choice of arranging these disks because the particle resonances localize the fields inside the particle, making the influence of the interparticle interaction negligible (unlike the lattice resonances, where the correct placement is of paramount importance). For simplicity, we chose to arrange the disks in a metasurface (MS) with a dense square lattice, with disk center-to center separation of 570 nm being limited only by our fabrication capabilities (namely, the height-to-width aspect ratio of both trenches and ridges was kept below 1). Then we characterize the fabricated MS by both optical measurements of reflectance and transmittance and by measuring a wavelength-dependent photocurrent (i.e., a responsivity spectrum). Our experiments agree well with simulations, and we demonstrate more than 10-fold improvement, compared with intact 105-nm-thin Ge film PD of the same size, revealing at the same time that the absorption enhancement and detector responsivity are increased due to the excitation of magnetic dipole resonances in Ge disks.

## 2 Simulations

We begin our investigation with numerical inspection of absorbing properties of single Ge disks on thin Ge film (the detailed explanation of the simulations is described in the Methods section). In accordance with the high technological demand, our aim is to realize a broadband detector in the targeted spectral range (1000–1600 nm), therefore, three target wavelengths of 1200, 1375, and 1575 nm, are taken, and for each of these wavelengths we vary the diameter and the height of the disk to obtain the best absorption, normalized to the cross-section area of the disk. The choice of these target wavelengths is dictated by the material absorptivity of Ge, which is high at short wavelengths, and approaching zero around 1600 nm (see measured refractive index of the fabricated amorphous Ge films in Supplementary Figure S1). We study both the total absorption and the absorption in the Ge layer caused by the presence of the disk. The latter is important, because the generated electron-hole pair in the disk experience weaker separating electric fields and, generally, has higher chances to recombine and therefore to reduce the responsivity of the PD.

**Figure 1:**
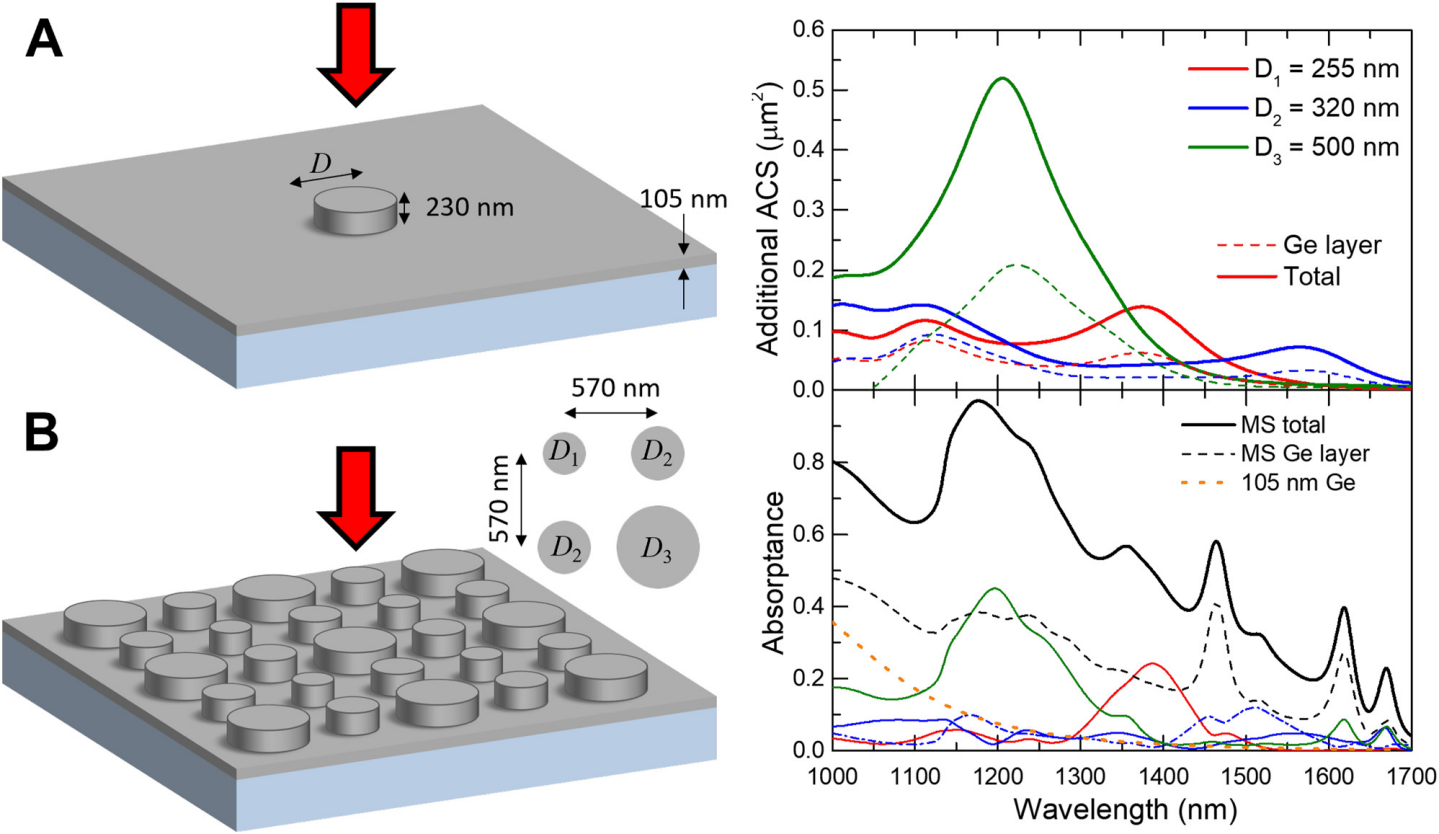
Simulated absorption results for individual disks and the MS. (A) Study of individual 230-nm-thick Ge disks on 105-nm-thin Ge layer, illuminated with a plane wave. Spectra of additional absorption cross-section (ACS), caused by the presence of the disk, are calculated for optimum disk sizes of 255, 320, and 500 nm in diameter (solid lines). Dashed lines indicate the partial absorption, calculated only in the Ge layer. (B) Study of absorptance of the MS, composed of three different disks, arranged in a square lattice and placed on Ge layer. Plot shows spectra of total absorptance (black solid line) and partial contributions, calculated inside each disk (lines of the same color as in (A)) and inside Ge layer (black dashed line). Here, two blue lines, solid and dash-dotted, correspond to two disks of the same diameter *D*_2_. For comparison, the absorptance spectrum of 105-nm-thin Ge layer without disks is shown (orange dotted lines).

We have found the optimum disk thickness of 230 nm and optimum disk diameters of 255 and 320 nm, providing largest absorption at design wavelengths of 1375 and 1575 nm, respectively (see Figure 1A). The best performance for the wavelength of 1200 nm was found for the disk diameter of ∼200 nm, which is smaller than the disk thickness and, thus, not feasible for our fabrication facilities, therefore the second-best choice of the disk with a diameter of 500 nm was chosen. To understand the origin of these resonances, we traced back to the case of isolated Ge disk in even air surrounding by gradually decreasing the Ge layer thickness and then, replacing the glass substrate with air (see Supplementary Figures S2–S7). Our first guess as well as the initial motivation was to exploit anapole states, featuring suppressed scattering and boosted local field (and, thus, absorption) [20–22]. To our surprise, the observed resonances originate exclusively from the magnetic dipole (MD) resonance. It appeared that disks at MD resonance feature nearly the same absorption as the ones at anapole state, but their footprint is smaller, which allows their denser arrangement. Additionally, the resonance associated with the anapole state disappears after addition of the Ge layer, perhaps due to the scattering into Ge layer (see Supplementary Figures S5–S7).

As expected, the absorption peaks are broad enough (Figure 1A), so that when the disks are combined in a MS, they should produce a uniform broadband absorptance spectrum. One can notice that the absorption cross-section (ACS) of each disk is several times larger than the cross-section area of the disk itself (see normalized ACS maps in Supplementary Figure S9), meaning that the absorptance close to unity is still achievable for the MS, even though each disk occupies only a small part of the whole surface.

A sketch of the designed MS is shown in Figure 1B, where all three different disks are combined in a square lattice with a center-to-center disk distance of 570 nm. To boost the otherwise weak absorption around the wavelength of 1575 nm (because it is close to the BG of Ge), the corresponding disk of diameter *D*_2_ = 320 nm was used twice in the MS cell. As expected, the absorptance spectrum of the MS is broadband, with only weakly pronounced peaks at *λ* ∼ 1200, 1375, and 1575 nm, corresponding to the resonances in each disk. There are, however, three pronounced peaks at *λ* ∼ 1475, 1625, and 1675 nm, which seems to stem from lattice resonances. This assumption is supported by the partial absorptance spectrum, calculated in the Ge layer, where these peaks are even more pronounced (compared with disk-related peaks). However, we don’t expect to see these lattice resonances in our experiment, because they require perfectly periodic structure (with periodically repeated elements of exactly the same size and shape) and near plane-wave illumination (since these resonances are angle-sensitive). But even without lattice resonances, our MS shows an improvement in absorption by a factor of 10 or higher, when compared to the absorption in the same 105-nm-thin Ge layer without disks (orange line in Figure 1B). Although total thickness of the MS is 335 nm, we believe it is fair to compare its performance with a bare Ge layer of 105 nm in thickness, because the dark current and corresponding noise is nearly unaffected by adding Ge disks (see Figure 4A). But even compared with a 335-nm-thick Ge layer, our MS is still superior in terms of absorption (see Supplementary Figure S9).

## 3 Experimental results and discussion

### 3.1 Sample fabrication

**Figure 2:**
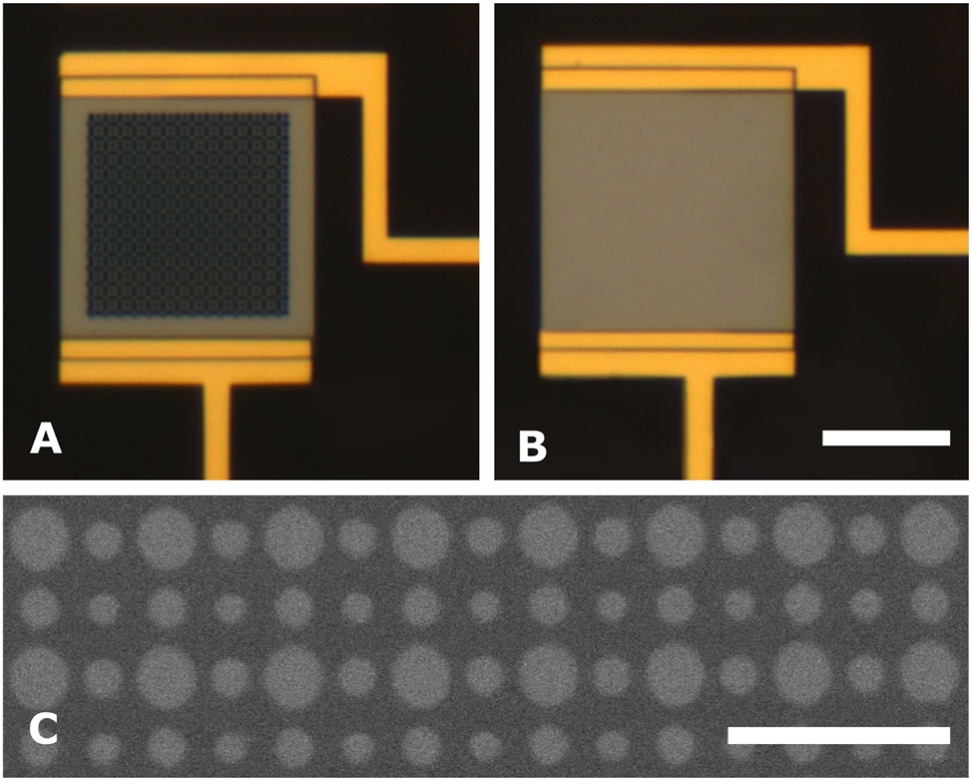
Images of the fabricated PDs. Optical images of the fabricated PDs, showing (A) the MS of Ge disks and (B) the 105-nm-thin Ge rectangle. Scalebar is 10 µm. (C) SEM zoomed-in image of the MS, clearly demonstrating the arrangement of Ge disks. Scalebar is 2 µm.

We fabricated two Ge PDs consisting of a 105-nm-thin Ge rectangle (20 μm × 22 μm in size) and gold (Au) electrodes attached to it. Then a MS of Ge disks (17 μm × 17 μm in lateral size) was fabricated on top of one Ge rectangle, with geometrical parameters obtained from simulations. Each PD was used for both optical and electrical characterization. The optical images of the PDs along with the zoomed scanning electron microscope (SEM) image of the MS are shown in Figure 2. The detailed information about fabrication process can be found in the Methods section.

### 3.2 Optical characterization

**Figure 3:**
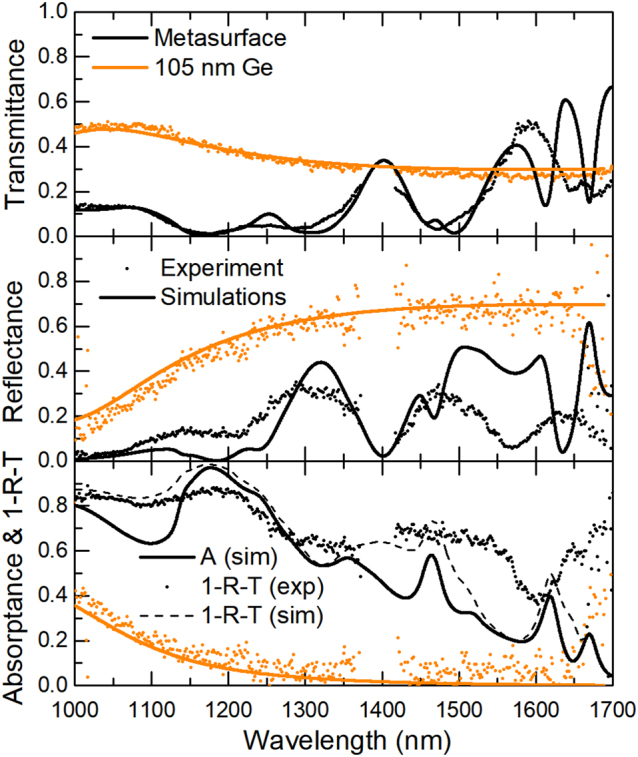
Numerically calculated transmission (*T*), reflection (*R*), extinction (1–*R*–*T*), and absorption (*A*) spectra (lines) for the 105-nm-thin Ge film (orange) and the MS of Ge disks (black), compared with measured spectra (dots).

To indirectly probe the absorption, we measured the transmission, *T*, and reflection, *R*, spectra of both PDs (Figure 3) and compared them with the simulated spectra. As can be seen, there is a fair agreement between experiments and simulations, especially for the transmission spectra, which is due to the lower numerical aperture (NA) of the illumination, used in the experiment, being closer to the simulations with the plane-wave incidence. As expected, features related to the lattice resonances near *λ* ∼ 1475 nm, 1625 nm, and 1675 nm are absent in the experiment (additional simulations with inclined incidence, demonstrating large angle sensitivity of these resonances, can be found in Supplementary Figure S10). The remaining difference between the experiment and the simulations can be attributed to the minor deviations in the fabrication (for example, because the walls of the fabricated Ge disks are not perfectly vertical). Additionally, we calculated the extinction, 1–*R*–*T*, and compared it to the absorption, *A*. The difference between the extinction and absorption spectra are diffraction losses, radiating in both the air and the glass substrate (see Supplementary Figure S9). It is worth mentioning that the wavelength gap near 1400 nm in the measurements is related to the experimental setup (losses in a fiber of our spectrometer) and not the sample.

### 3.3 Photocurrent measurements

**Figure 4:**
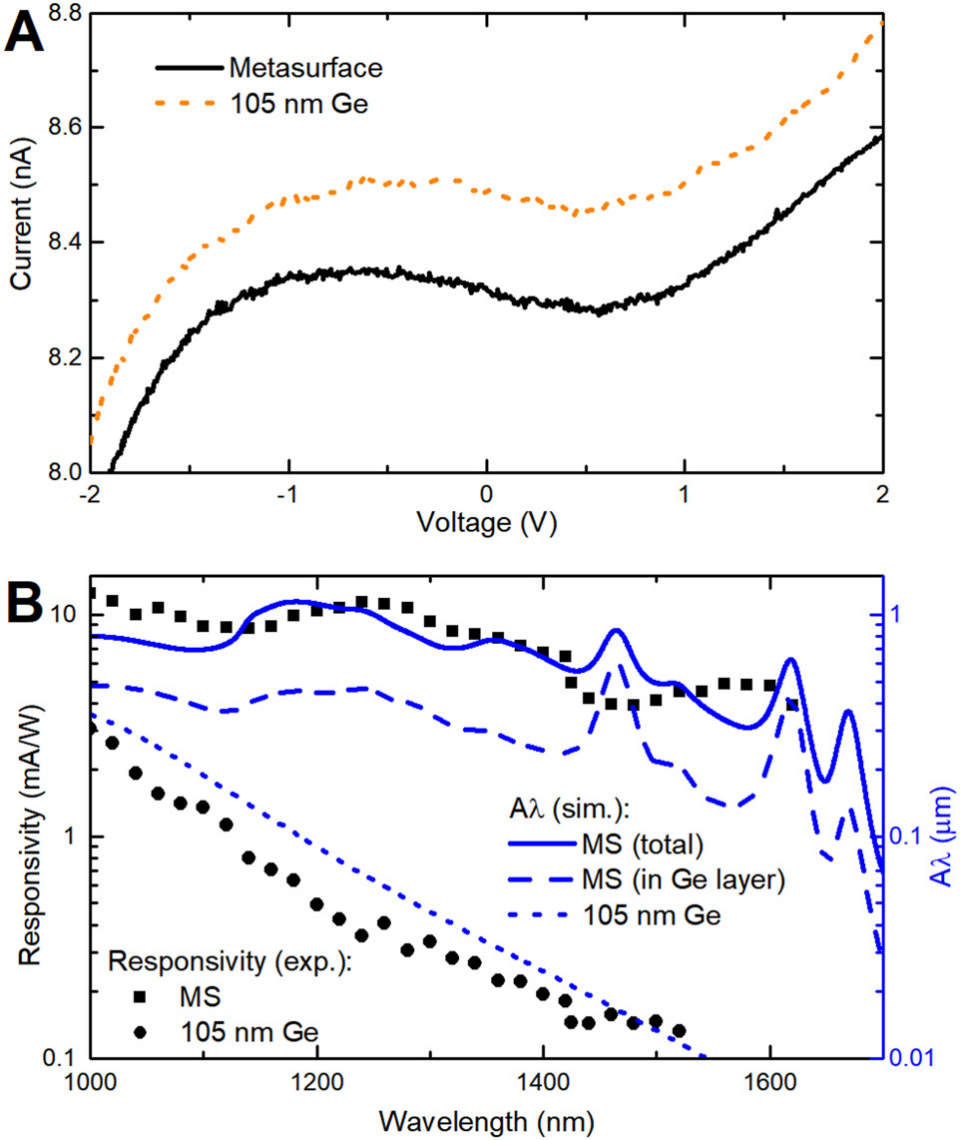
Performance of the PDs. (A) Current–voltage characteristics of fabricated PDs without incident light. (B) Measured photoresponsivity of both PDs (symbols), compared with the simulated absorptance spectrum, multiplied by the wavelength (lines). Dashed line corresponds to the partial contribution to the absorption, calculated only inside 105-nm-thin Ge layer of the second PD with the Ge disk MS.

Next, we proceed with measuring the current–voltage characteristics of fabricated PDs without incident light (Figure 4A). The nonlinear behavior is cognate to the Schottky contact of the Ge layer and gold electrodes. One can note that even in the absence of the external bias voltage the current through both PDs is higher than zero, and there is a vertical shift between the two curves. The nonzero current is caused by the asymmetry of the Ge/Au contacts (hence asymmetry of the electric fields in those Schottky contacts) due to the imperfection of fabrication, an asymmetry that results in an internal electric field. The vertical shift, consequently, is a result of a slight variation of that field from one detector to another, which however does not affect the overall behavior of the PDs (Supplementary Figure S11). An important point here is that the dark current for the structured PD is nearly the same as for the thin film PD, which shows the huge advantage of nanostructuring the material instead of simply increasing the absorbing volume. The measured photoresponsivity of two PDs at different wavelengths is shown in Figure 4B and compared with the absorptance spectra, multiplied by the wavelength (to correlate it with the responsivity, which is proportional to the number of absorbed photons). The bias voltage was set to 4 V resulting in approximately 12 nA dark current for both PDs (additional measurements of the photocurrent at various incident powers of light and at different bias voltages are shown in Supplementary Figure S12). In essence, the Ge MS PD is producing a three-fold enhancement of the responsivity in the beginning of the studied wavelength range, which grows up with the increase of the wavelength, reaching about a hundred-fold enhancement at the wavelength of 1550 nm. Though the experimental spectra resemble the simulated ones as far as broad features are concerned, the peaks in the simulated absorption spectrum near *λ* ∼ 1475 nm and 1625 nm are not reproduced. This difference originates from the circumstance that these peaks are associated with the lattice resonances, which are pronounced in the simulations conducted for the plane-wave incidence but smoothed out in our experiment with the illumination by a focused beam (see the angular dependence of the absorption spectrum in Supplementary Figure S10). The focused illumination in the experiment was crucial to avoid illuminating gold contacts, which will otherwise add the contribution of the photocurrent from the Schottky contact.

## 4 Conclusions

In this study we demonstrate the concept of enhancing the light absorption in a thin photodetection layer by adding high-index nanostructures on its surface, which both localize the fields (inside the particle and the photodetection layer) and scatter the light into the layer. We used an all-Ge semiconductor platform with Ge as the material for both, photodetection layer and disks on top. We concluded that the optimum performance is achieved at the resonance associated with the excitation of the magnetic dipole. This resonance can easily be tuned by changing the disk diameter, thereby allowing one to realize flat broadband absorption by utilizing differently sized disks. In our demonstration we selected three disk sizes (namely, 255 nm, 360 nm, and 500 nm in diameter), which correspond to the resonances at the designed wavelengths of 1375 nm, 1575 nm, and 1200 nm, correspondingly. The disks are arranged in a square lattice with the center-to-center distance of 570 nm, in which the 360-nm-diameter disk was used twice to boost the absorption at the wavelength of 1575 nm. The square lattice arrangement was used to simplify simulations and fabrication. Alternatively, one can arrange disks randomly (the denser the better), with disk diameters chosen randomly from a certain statistical distribution determined in accordance with a desired absorption spectrum. We have demonstrated the improvement in photocurrent by a factor, ranging from 3 to ∼100 in the wavelength range 1000–1550, over the device without disks (i.e., containing only 105 nm Ge layer), while maintaining essentially the same dark current.

Our design features the following practical advantages: (1) disks can be made of non-absorbing high-index material (e.g., silicon) to avoid light absorption inside the disks, since the latter contribution to the photocurrent is weak; (2) the photodetection layer does not require patterning, therefore it can be made of a high-quality material (e.g., InGaAs), keeping intact its quality (that might otherwise be affected by nanostructuring); (3) the enhancement in absorption is due to the individual particle resonances (e.g., the magnetic dipole resonance in disks), alleviating the fabrication tolerances considerably. The latter circumstance suggests the disk fabrication by etching through the mask, defined by a cheaper UV-lithography technique, or even produced by coating the surface with polystyrene beads.

## 5 Methods

### 5.1 Simulations

All simulations were conducted with finite element method implemented in COMSOL v5.6 software, with refractive index of glass assumed to be 1.45, and Ge refractive index taken from measurements (Supplementary Figure S1). First, we have simulated individual disks, placed inside a domain, surrounded with perfectly matched layers (PML), in a scattered field formulation, where the analytically calculated field of the normally incident plane wave and its reflection from the substrate is taken as a background field. Due to the symmetry of the incident field and the geometry, we simulated only one quarter of the domain, with corresponding perfect electric conductor and perfect magnetic conductor boundaries placed in symmetry planes. The domain was a sphere with a radius being equal to the free-space wavelength, *λ*, for the case of disks in air or on glass. For simulations with Ge layer (Figure 1A and Supplementary Figures S5–S7, S9), the domain was changed to a cylinder (coaxial with the disk) with a radius of 2 µm and a height of 2*λ*. In this case the absorption in Ge layer was underestimated by calculating only the absorption inside the calculation domain (volume integral of the total power dissipation density in the Ge layer), since the power carried away by guided TE and TM modes, which is eventually absorbed in Ge layer for infinitely large domain is neglected (see Supplementary Figure S8). The absorption of the background field in Ge layer was subtracted. Next, we conducted simulations of the MS, with a square unit cell of *P* = 1140 nm of side length and periodic boundary conditions (on sides), while the top and bottom boundaries were placed one wavelength away from the Ge layer, being defined as active (input) and passive ports, correspondingly, with automatic calculations of the diffraction orders (which is happening in glass for *λ* < *n*_glass_*P = 1140*1.45 = 1653 nm, while diffraction into air is only present for *λ* < 1140 nm). Note that due to the reciprocity principle, the direct transmission is the same, independent of the choice of the input port (which is not the case for absorption). Here, the absorption was simply calculated as a volume integral of the total power dissipation density.

### 5.2 Fabrication

Fabrication of the device relies on a multistep lithographic process using the mix-and-match technique for lithographic overlay. First, connecting electrodes are patterned onto a glass chip by optical lithography, metal deposition (5 nm Titanium, Ti, and 50 nm Au), and lift-off. Subsequently windows for thin-film (105 nm) Ge are made by electron-beam lithography (EBL) at an acceleration voltage of 30 keV in 300 nm PMMA resist and 60 nm thick conductive polymer (AR-PC 5090.02) to dissipate charge accumulation on an insulating surface. After development, a 105 nm thick Ge layer is deposited by thermal evaporation and subsequent 12 h lift-off in acetone. Next, we fabricated gold electrodes with the same technique. The parameters for the PMMA and conductive polymer are the same, and after the EBL and development, 2 nm of Ti and 120 nm of gold is deposited by the thermal evaporation. Finally, for the fabrication of 230-nm-thick disks we still used the same routine with the only difference in PMMA thickness which was 600 nm in this case. The alignment between the different lithography steps is performed manually using additionally fabricated markers.

### 5.3 Spectroscopy

The transmission and reflection spectra were measured by Ocean Insight NIRQuest +1.7 spectrometer with InGaAs linear array. As a light source we used SuperK EXTREME supercontinuum laser from NKT Photonics (ranging from 400 to 2400 nm), where the visible part of the spectrum was filtered out by long-pass filter. The schematic of the setup is displayed in Supplementary Figure S13. In both reflection and transmission measurements the illumination was realized with loosely focused beams covering only the area of the fabricated MS (approximately 15 μm in diameter). For the collection, a high NA objective was used. For the references we measured the transmission through and the reflection from the PDs substrate (borosilicate glass), and then recalculated absolute values, assuming the glass refractive index of 1.45.

### 5.4 Photocurrent measurements

To measure the photocurrent, we used several tunable laser sources to cover the desired spectra, namely SuperK EXTREME with SELECT tunable narrowband filter (spectral range of 900–1400 nm), tunable CW laser New Focus TLB-6500-h-es (1425–1525 nm), and tunable CW laser New Focus TLB-6600 (1525–1630 nm). The PDs were illuminated in the same reflection configuration as shown in Supplementary Figure S13. Electrical connections were realized with touching pads. All the measurements were carried out at room temperature; also, the samples (both PDs) sustained functionality over a six-month period of storage under ambient conditions.

## Supplementary Material

Supplementary Material Details
