# Supplementary material for: Germanium metasurface assisted broadband detectors
Source: Nanophotonics. 2023 May 11;12(12):2171–7. doi: 10.1515/nanoph-2023-0116 (PMC11502065; doi:10.1515/nanoph-2023-0116)
Supplement: Supplementary file 1 — Supplementary Material Details [file j_nanoph-2023-0116_suppl_001.pdf]

Supporting Information for

Germanium metasurface assisted broadband detectors

Torgom Yezekyan, Vladimir A. Zenin, Martin Thomaschewski, Radu Malureanu, Sergey I. Bozhevolnyi*

AUTHOR ADDRESS. Centre for Nano Optics, University of Southern Denmark, Campusvej 55, DK-5230 Odense M, Denmark

*Corresponding author: ty@mci.sdu.dk

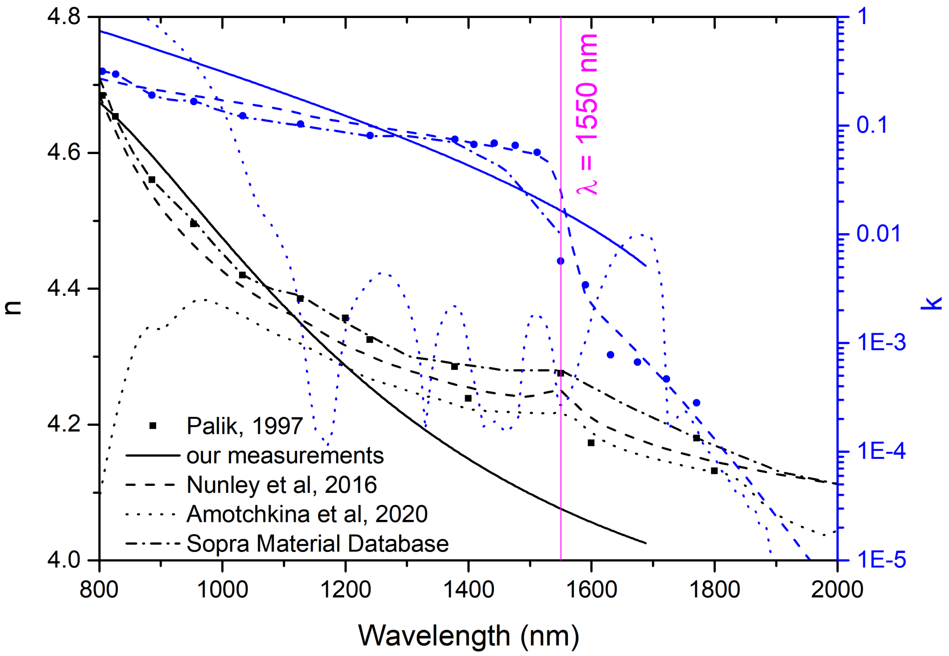

Figure S1. Refractive index of the fabricated amorphous Ge, measured by standard reflectometry (FilmTek 4000TM), and compared with reported values in [1-4].

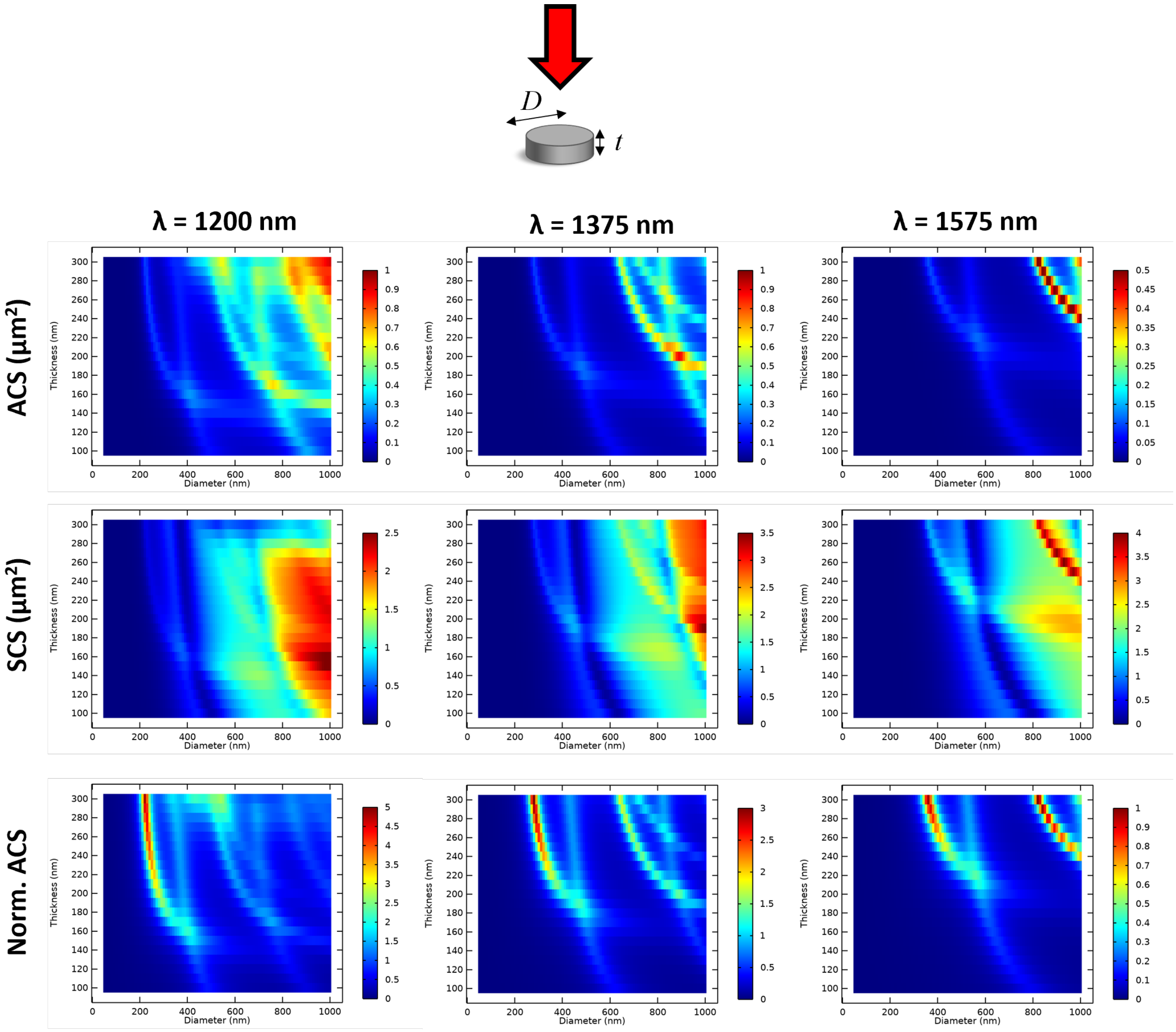

Figure S2. Simulations of an individual Ge disk in even surrounding (vacuum) with varied disk thickness *t* and diameter *D*. Absorption cross-section (ACS), scattering cross-section (SCS), and ACS, normalized to the disk area of $\pi D^2/4$, calculated at three different wavelengths: 1200, 1375, and 1575 nm.

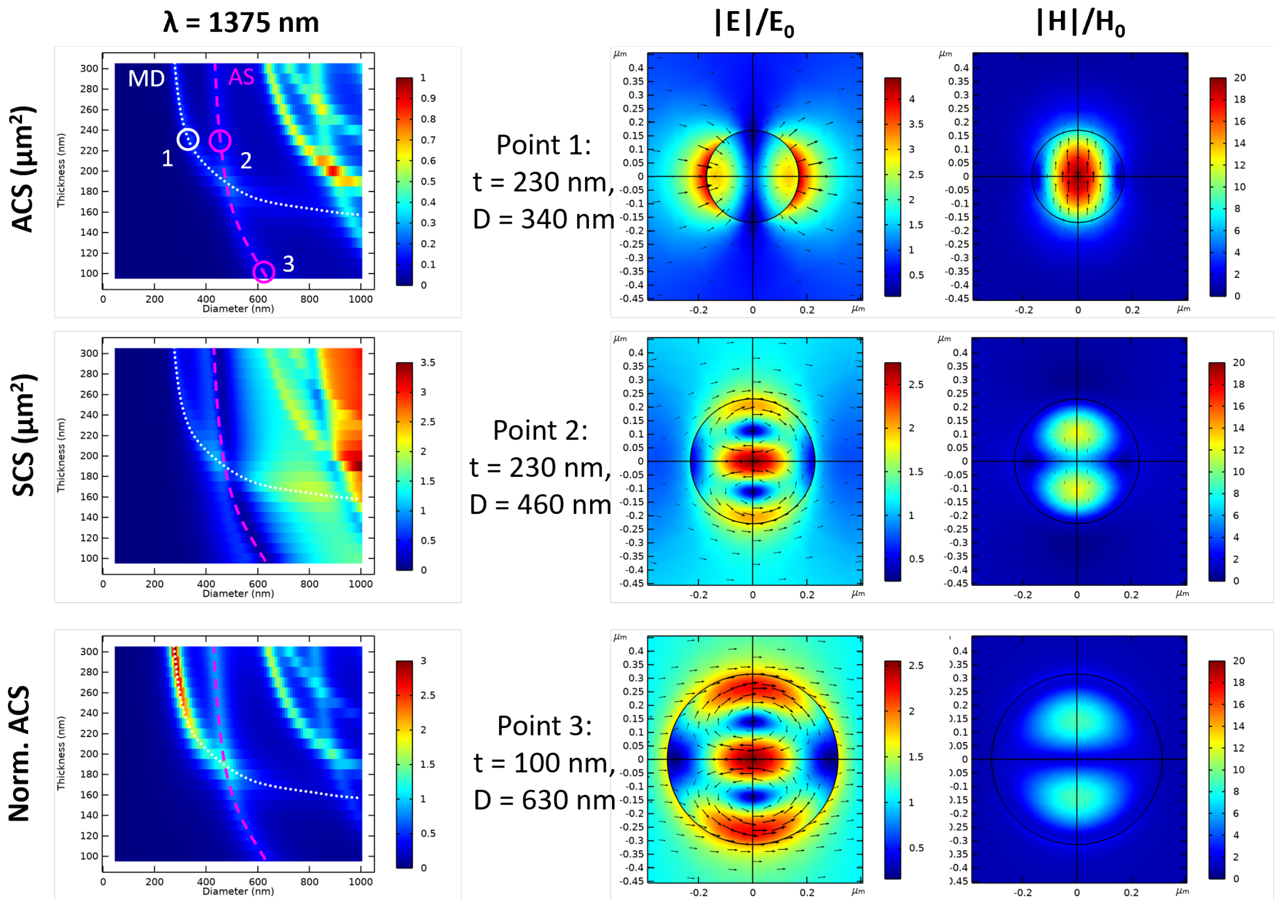

Figure S3. Closer investigation of optical resonances at λ = 1375 nm for Ge disk in even surrounding (vacuum). White dotted line and magenta dashed line indicates local maxima in ACS, which was found to be magnetic dipole (MD) and anapole state (AS) resonances. Note that scattering is near local minima for AS, as expected. One point in MD line (with t = 230 nm and D = 340 nm) and two points in AS line (point 2 with t = 230 nm, D = 460 nm; and point 3 with t = 100 nm and D = 630 nm) were selected for investigation of induced fields. Middle and right columns show amplitude of electric and magnetic fields, normalized to the incident field magnitudes, mapped at the center of the disk. Electric field distributions for points 2 and 3 can be unambiguously attributed to the AS [5-7], while magnetic field distribution for point 1 proves that it is the MD resonance, where magnetic dipole is oriented along the magnetic field of the incident plane wave. Thus, it is obvious that MD resonance is superior to AS resonance in normalized ACS for disk thickness above ~200 nm.

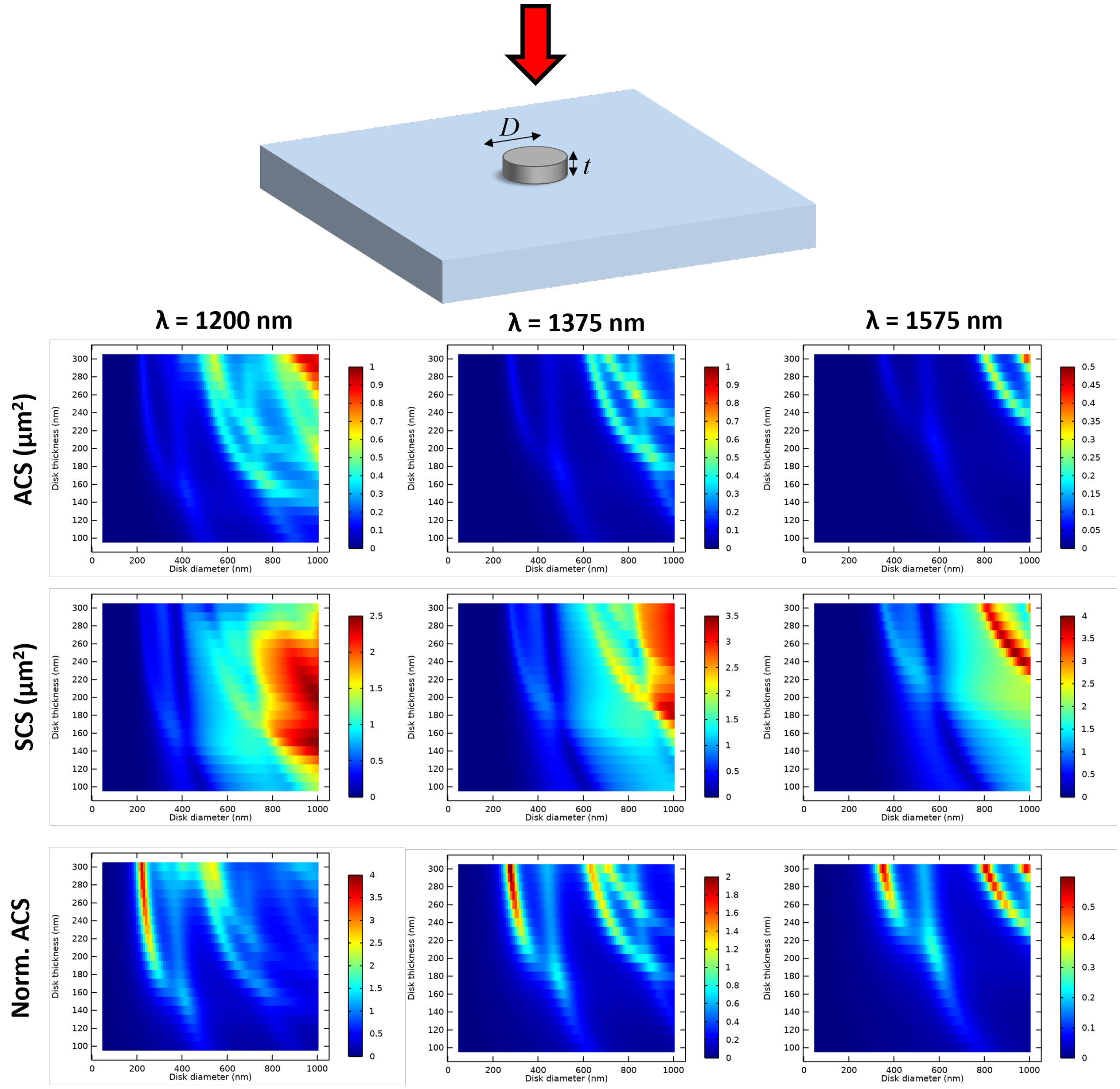

Figure S4. Simulations of an individual Ge disk, placed on semi-infinite glass substrate (n = 1.45) with varied disk thickness and diameter. Absorption cross-section (ACS), scattering cross-section (SCS), and ACS, normalized to the disk area of $\pi D^2/4$, calculated at three different wavelengths: 1200, 1375, and 1575 nm.

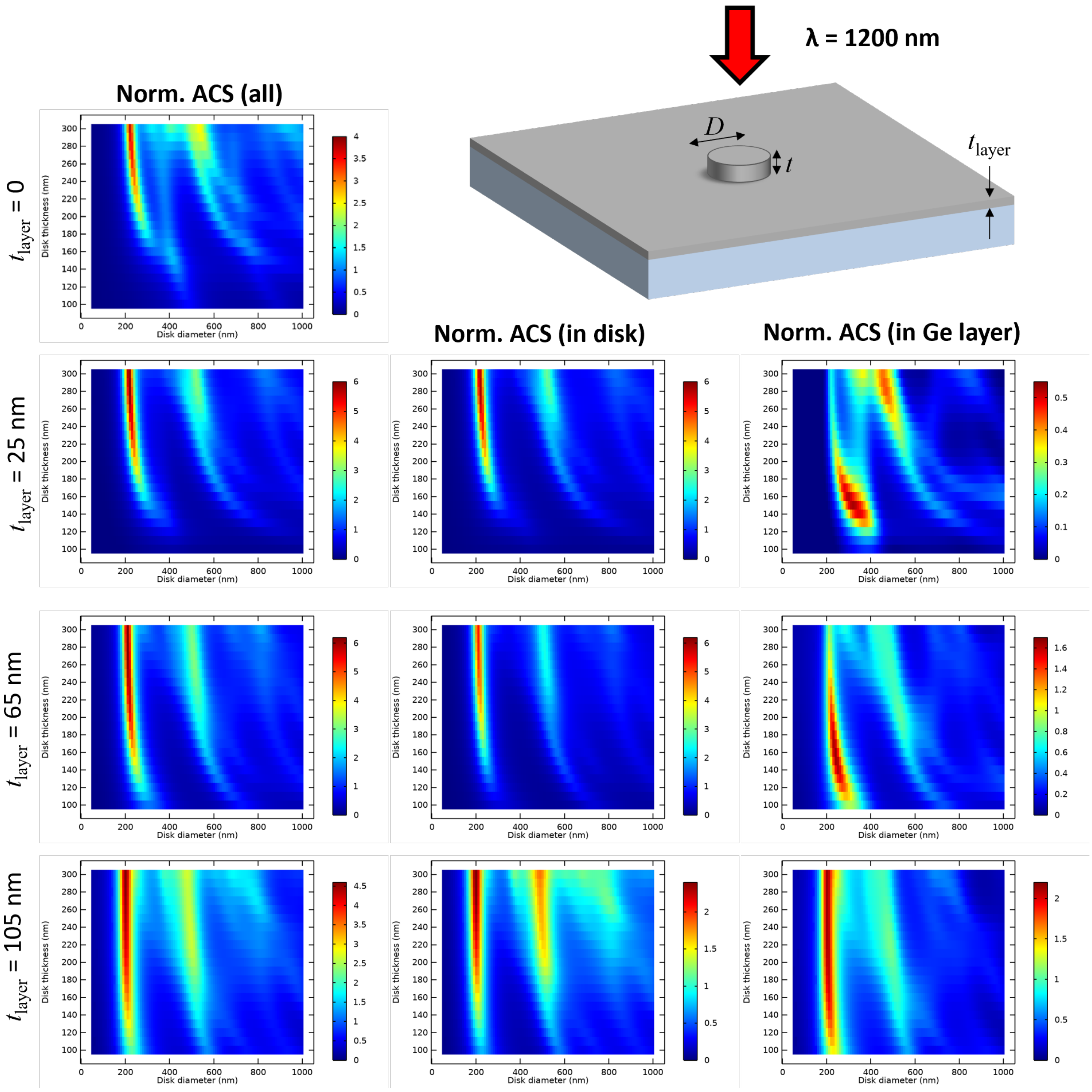

Figure S5. Simulations of an individual Ge disk, placed on semi-infinite glass substrate (n = 1.45), covered with Ge layer of thickness t_{layer}. Maps shows absorption cross-section (ACS), normalized to the disk area of $\pi D^2/4$ and calculated in both disk and Ge layer (left column) and only in Ge layer (right column). The absorption of Ge layer without any disk was subtracted. The incident plane-wave wavelength is 1200 nm. Note the disappearance of AS-related resonance when Ge layer is added. The increase of Ge layer thickness leads to the increase of absorption (both total and partial contribution from Ge layer). For each fixed Ge thickness the total absorption is increasing with the increase of the disk thickness, but for the partial contribution from the Ge layer there is an optimal disk thickness. The optimum disk diameter is ~200 nm, which violates our fabrication constraints, therefore we have chosen disk diameter of 500 nm, corresponding to the higher-order resonance.

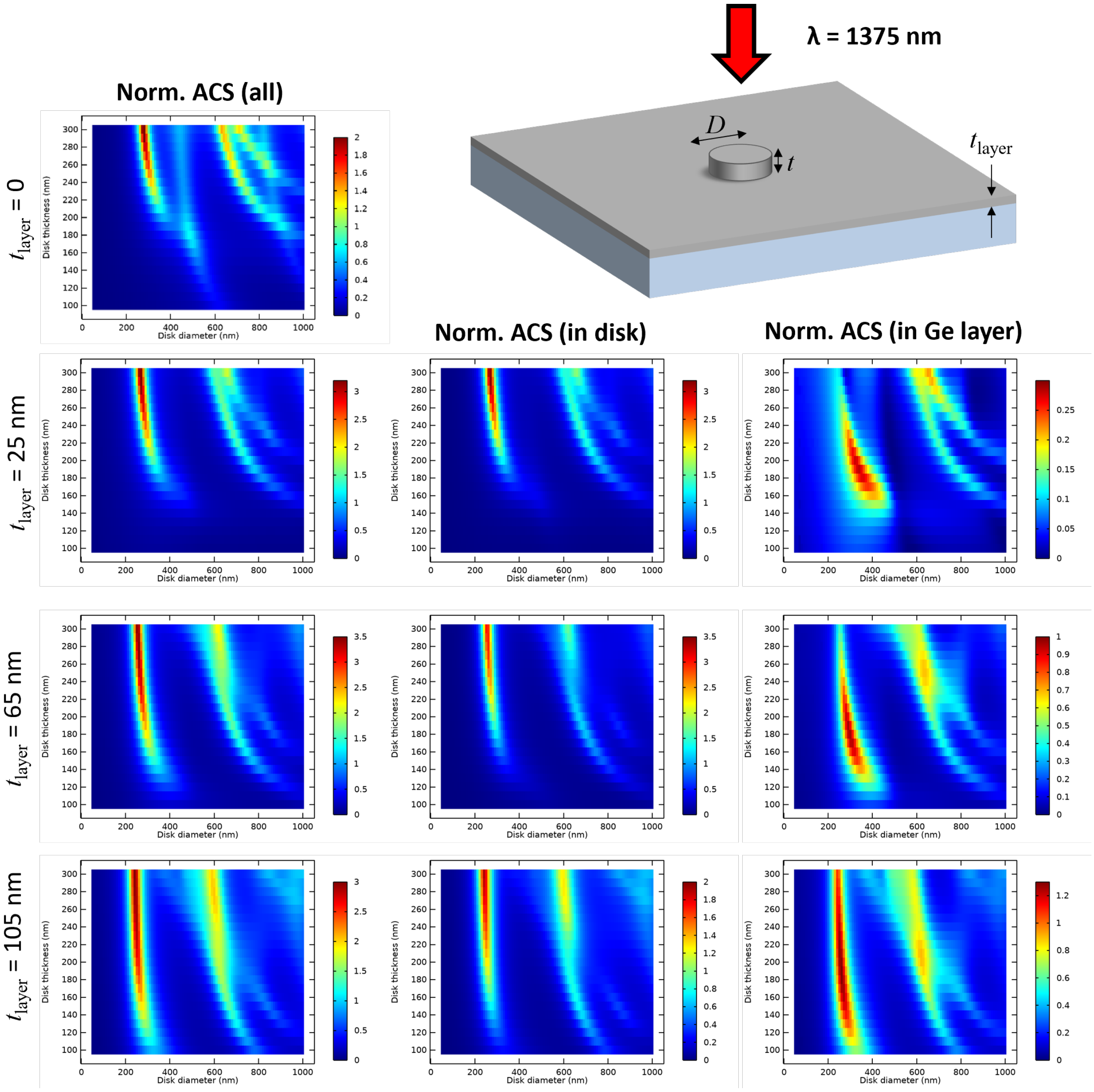

Figure S6. Same as Figure S5, but for the incident wavelength of 1375 nm. Trends are the same as for the incident wavelength of 1200 nm. Note that for the Ge layer thickness of 105 nm the disk thickness of 230 nm seems to be optimal to provide near-maximum values for both total absorption and partial contribution from the Ge layer.

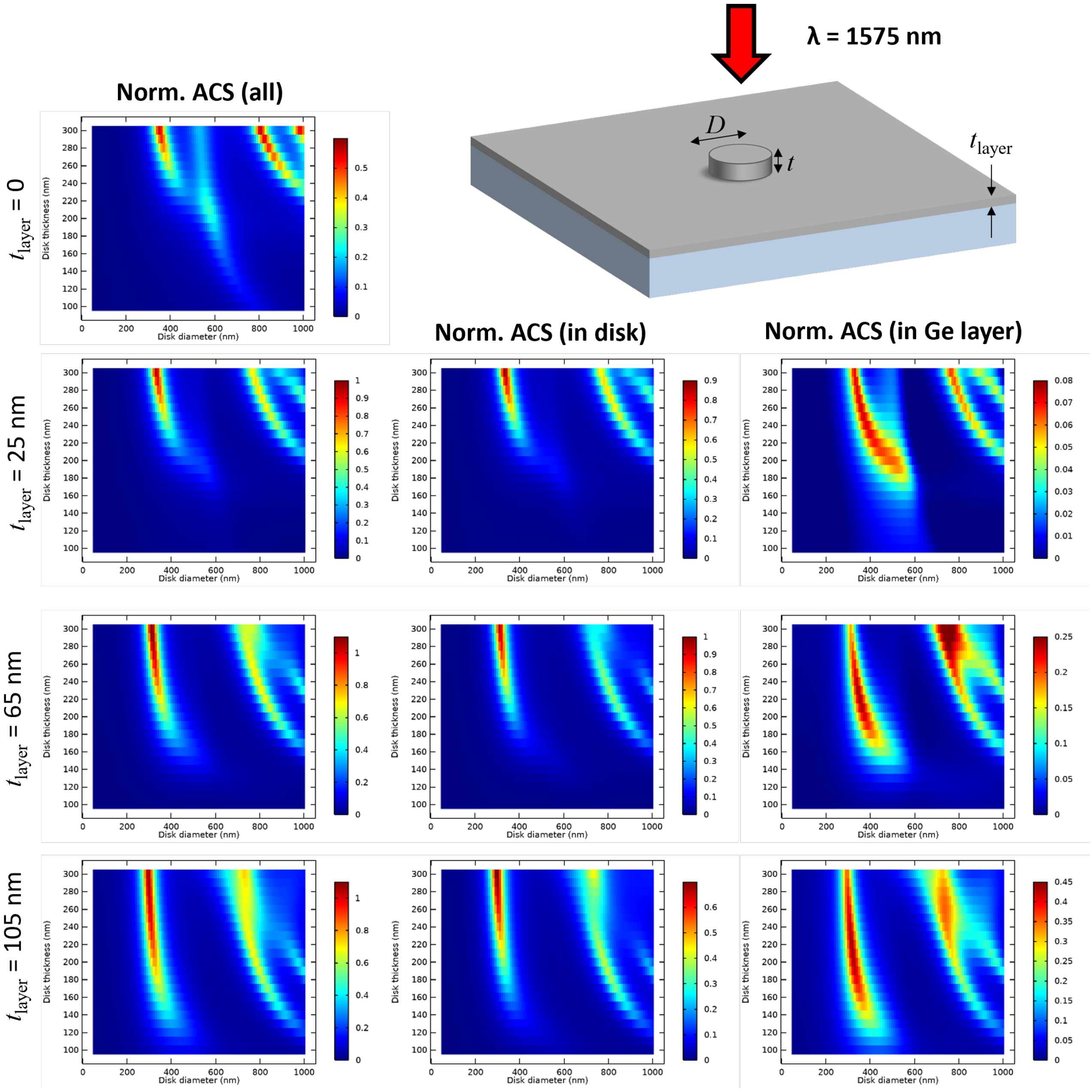

Figure S7. Same as Figure S5, but for the incident wavelength of 1575 nm. Trends are the same as for the incident wavelength of 1200 nm. Note that for the Ge layer thickness of 105 nm the disk thickness of 230 nm seems to be optimal to provide near-maximum value for the partial contribution to the absorption from the Ge layer.

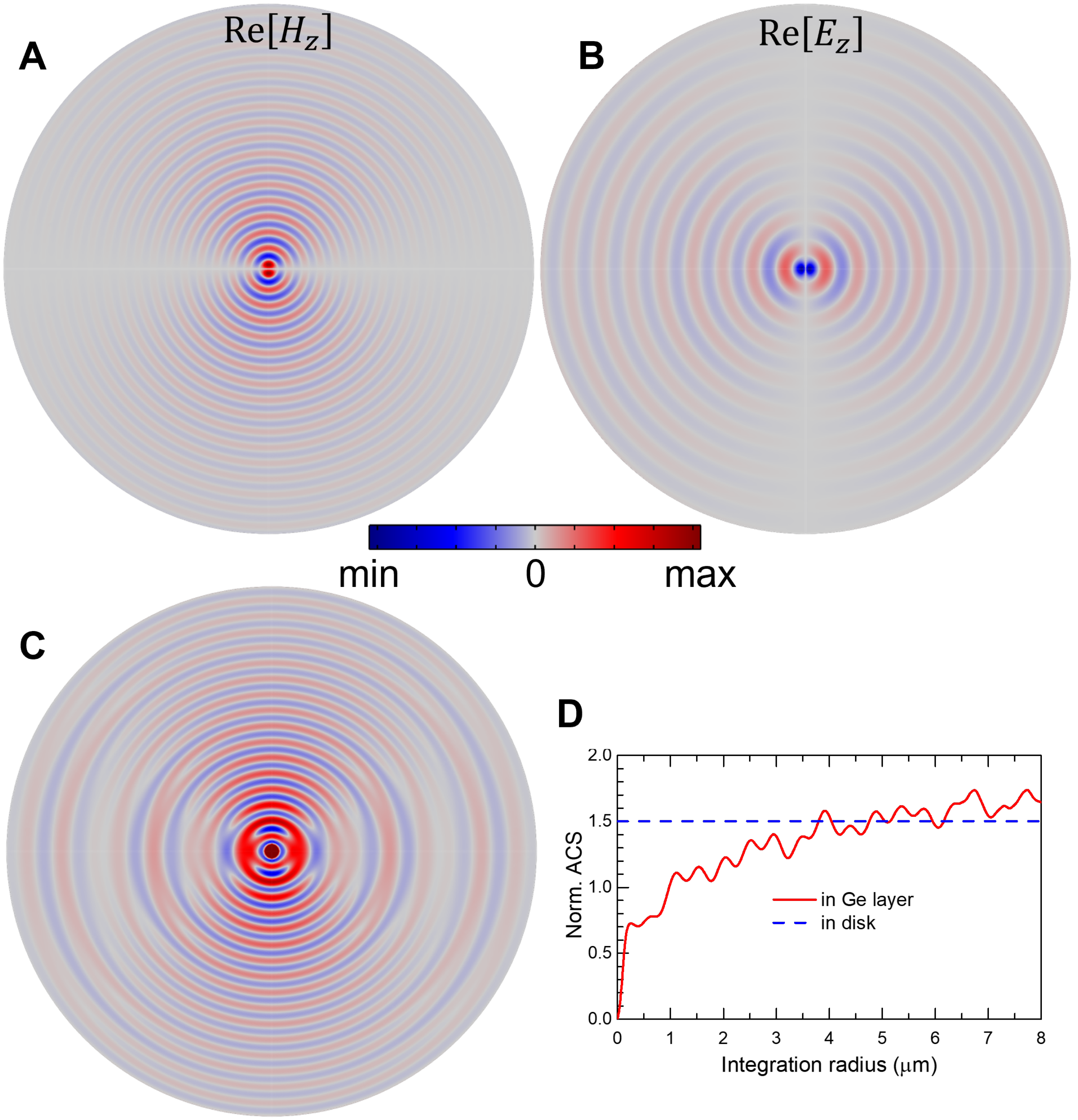

Figure S8. Large domain simulation of a resonant Ge disk at the wavelength of 1375 nm (disk thickness is 230 nm, diameter is 255 nm, and Ge layer thickness is 105 nm). **A, B** Distribution of the real part of the normal component of **A** magnetic and **B** electric field on the Ge-glass interface, illustrating scattering into guided TE and TM modes, respectively. **C** Representation of the absorption density in Ge layer, calculated as $\frac{1}{2}\omega\varepsilon_0\,\mathrm{Im}\,\varepsilon_{\mathrm{Ge}}/I_0\int_0^{t_{\mathrm{layer}}}(|E|^2-|E_0|^2)dz$, where E_0 and I_0 are the electric field and the intensity of the background field. **D** The influence of the domain size (done by integrating within coaxial cylinder of varied radius) on the normalized ACS in Ge layer, with the absorption of the background field being subtracted. The oscillations are caused by the interference of scattered TE and TM modes with the background field. Note that chosen domain radius of 2 μm for other simulations underestimates ACS in Ge layer by about 50%.

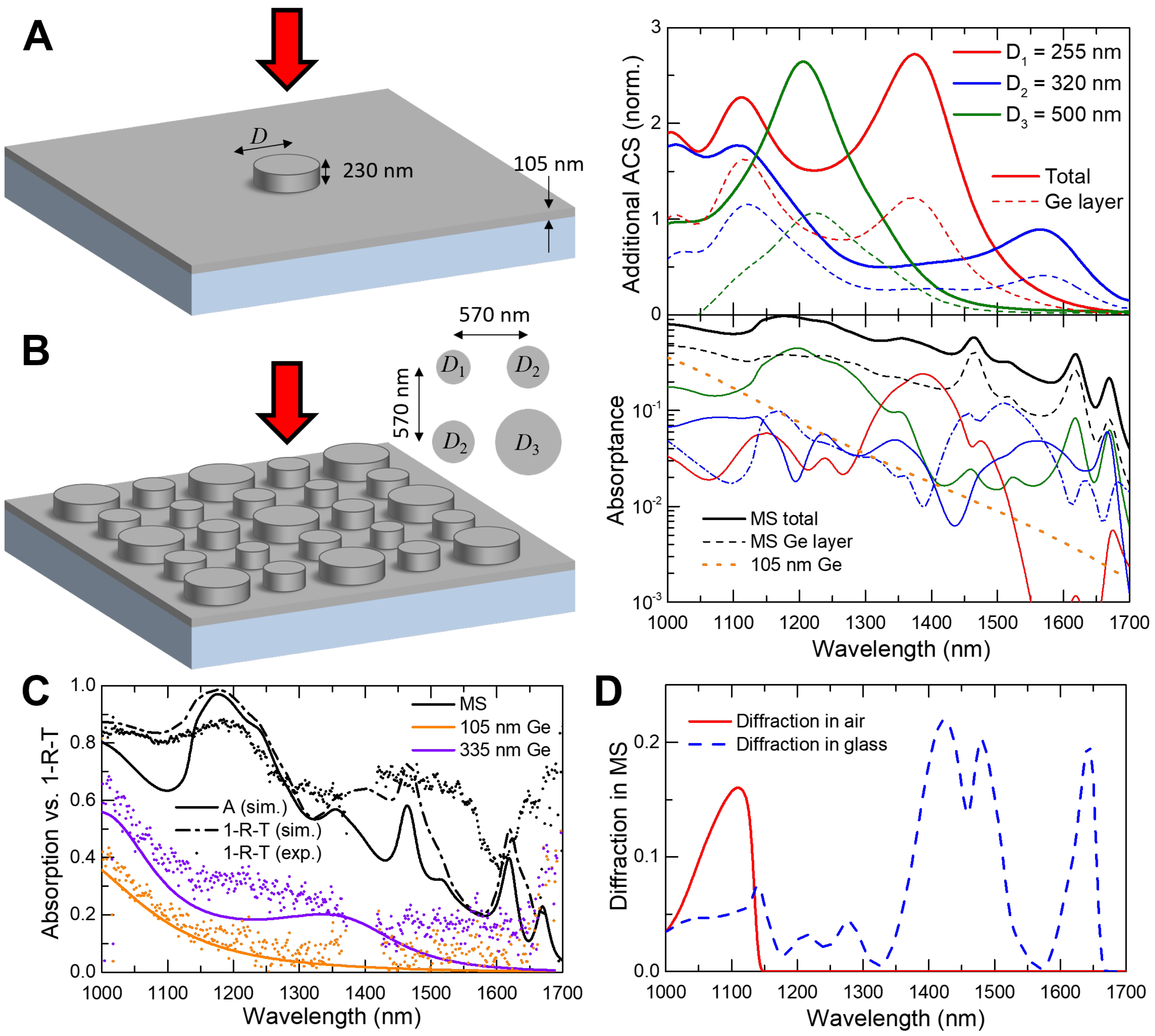

Figure S9. A, B: same as Figure 1 in the main text, but with **A** additional ACS, normalized to the disk area of $\pi D^2/4$; and **B** absorptance, plotted in log scale. **C** Comparison of simulated absorption A with measured extinction 1-R-T for the MS (black) and flat Ge of thickness 105 nm (orange) and 335 nm (violet). The difference for the MS is caused by the diffraction. **D** Diffraction in air and glass, calculated for the MS. Diffraction into air is only present for $\lambda < P$ = 1140 nm, while diffraction into glass is limited by $\lambda < n_{glass}P$ = 1.45*1140 = 1653 nm. Note that both transmission and diffraction into glass can be partially harvested in the MS by an addition of buried reflecting surface.

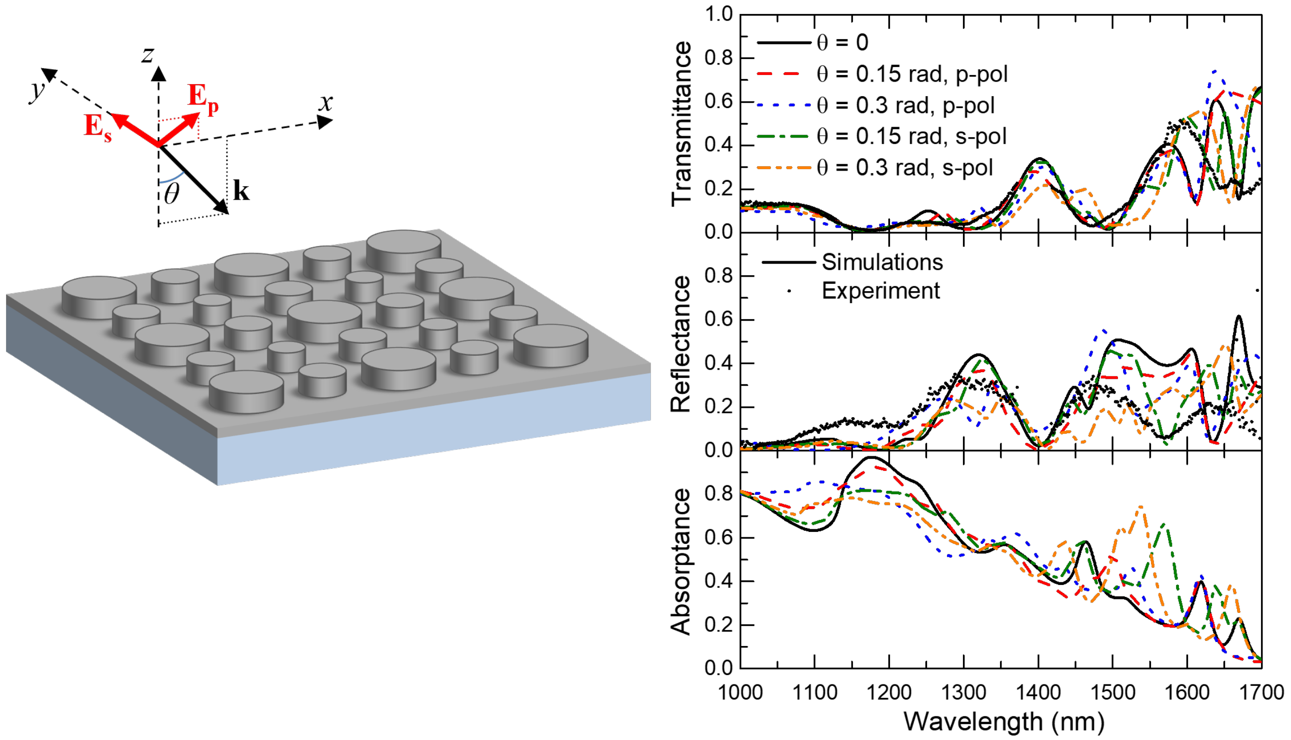

Figure S10. Influence of the incident angle on the optical performance of our MS. The MS is irradiated with either s- or p-polarized plane wave, incident at an angle of θ to the normal (see a sketch on the left). As expected, lattice resonances near $\lambda \sim 1475$ nm, 1625 nm, and 1675 nm are angle-dependent (especially for the s-polarization). On the contrary, the peaks near $\lambda \sim 1400$ nm in the transmission and near $\lambda \sim 1300$ nm in the reflection re more robust to the variation of angle, which results in good agreement with the experiment. Finally, the absorption spectra for different incident angles are similar and broadband, demonstrating the robustness of our design for practical applications.

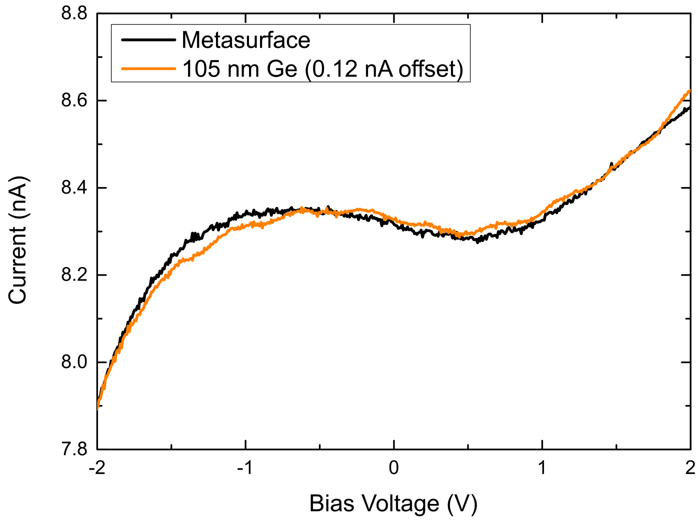

Figure S11. Current-voltage characteristics of fabricated PDs without incident light; same as the Fig. 4A of the main text, but with the curve for 105 nm Ge film being offset by 0.12 nA to show the similar behavior of the two PDs.

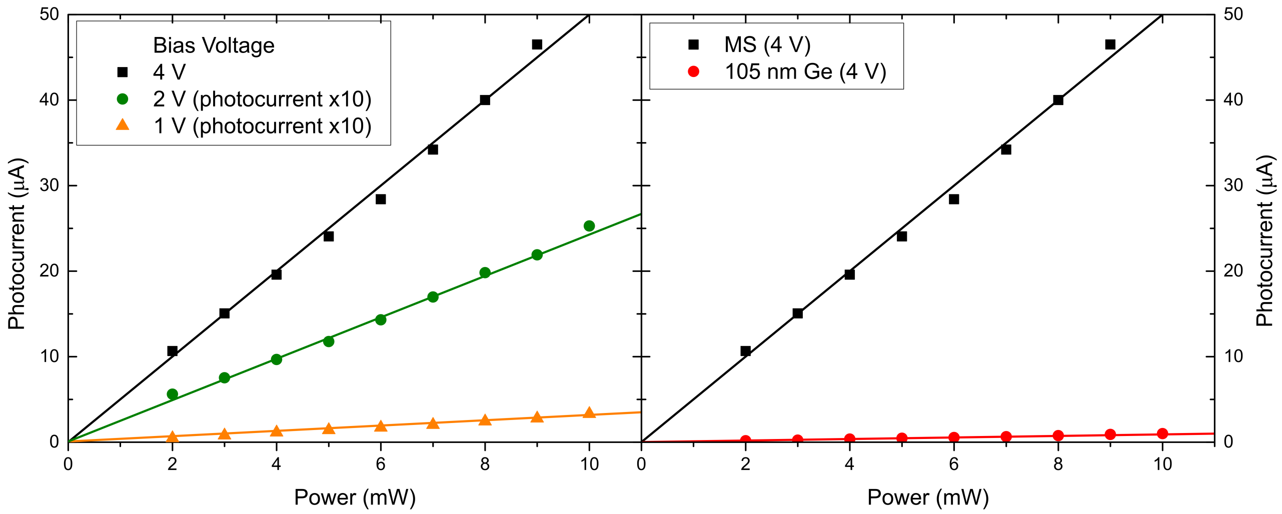

Figure S12. Photocurrent of the MS PD as a function of light power for various applied bias voltages (the left plot); compared to the photocurrent of the 105 nm thick Ge thin film PD (right plot). The incident light wavelength is set to 1550 nm. Note that in the left plot the photocurrent is multiplied by 10 for low bias voltages (1 V, 2 V).

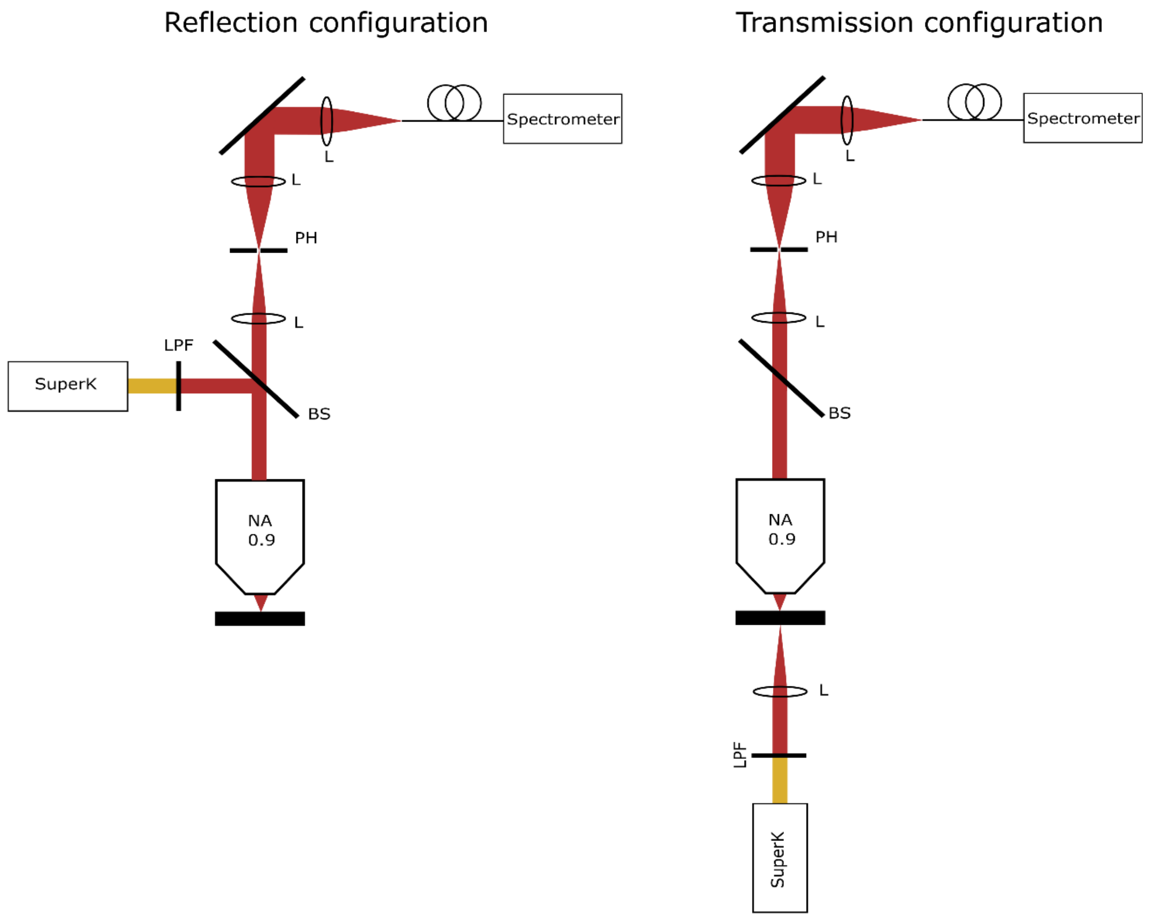

Figure S13. Schematics of the reflection and transmission configurations used during spectroscopy. Here LPF denotes low-pass filter, BS – beam splitter, L – lens, PH – pinhole. In both configurations the beam size and lens focal length (in transmission configuration) are chosen to achieve low NA of illumination (estimated as NA ~ 0.3 in reflection and NA ~ 0.15 in transmission), but at the same time to have the focal spot being smaller than the photodetector area. Additionally, a set with two lenses and a pinhole were used for spatial filtering. Finally, the light was coupled to a multimode fiber with a core diameter of 200 µm connected to the spectrometer.

References

1. T. Amotchkina, M. Trubetskov, D. Hahner, and V. Pervak, "Characterization of e-beam evaporated Ge, YbF 3, ZnS, and LaF 3 thin films for laser-oriented coatings," *Applied Optics,* vol. 59, no. 5, pp. A40-A47, 2020.
2. T.N. Nunley, N.S. Fernando, N. Samarasingha, et al., "Optical constants of germanium and thermally grown germanium dioxide from 0.5 to 6.6 eV via a multisample ellipsometry investigation," *Journal of Vacuum Science & Technology B, Nanotechnology and Microelectronics: Materials, Processing, Measurement, and Phenomena,* vol. 34, no. 6, pp. 061205, 2016.
3. E.D. Palik, *Handbook of optical constants of solids,* Vol. 3. Academic press (1998).
4. *Sopra Material Database*. Available from: https://www.filmetrics.com/refractive-index-database/Ge/Germanium.
5. Y. Yang and S.I. Bozhevolnyi, "Nonradiating anapole states in nanophotonics: from fundamentals to applications," *Nanotechnology*, vol. 30, no. 20, pp. 204001, 2019.

6. Y. Yang, V.A. Zenin and S.I. Bozhevolnyi, "Anapole-assisted strong field enhancement in individual all-dielectric nanostructures," *ACS Photonics*, vol. 5, no. 5, pp. 1960-1966, 2018.
7. T. Yezekyan, V.A. Zenin, J. Beermann, and S.I. Bozhevolnyi, "Anapole states in gap-surface plasmon resonators," *Nano Letters*, vol. 22, no. 15, pp. 6098-6104, 2022.
